# Next move in movement disorders: neuroimaging protocols for hyperkinetic movement disorders

**DOI:** 10.3389/fnhum.2024.1406786

**Published:** 2024-08-30

**Authors:** Jelle R. Dalenberg, Debora E. Peretti, Lenny R. Marapin, A. M. Madelein van der Stouwe, Remco J. Renken, Marina A. J. Tijssen

**Affiliations:** ^1^Expertise Center Movement Disorders Groningen, University Medical Center Groningen, Groningen, Netherlands; ^2^Department of Neurology, University of Groningen, Groningen, Netherlands; ^3^Laboratory of Neuroimaging and Innovative Molecular Tracers, Geneva University Neurocentre and Faculty of Medicine, University of Geneva, Geneva, Switzerland; ^4^Cognitive Neuroscience Center, Department of Biomedical Sciences of Cells and Systems, University Medical Center Groningen, Groningen, Netherlands

**Keywords:** hyperkinetic movement disorders, fMRI, FDG PET, tremor, myoclonus, dystonia, myoclonus-dystonia

## Abstract

**Introduction:**

The Next Move in Movement Disorders (NEMO) study is an initiative aimed at advancing our understanding and the classification of hyperkinetic movement disorders, including tremor, myoclonus, dystonia, and myoclonus-dystonia. The study has two main objectives: (a) to develop a computer-aided tool for precise and consistent classification of these movement disorder phenotypes, and (b) to deepen our understanding of brain pathophysiology through advanced neuroimaging techniques. This protocol review details the neuroimaging data acquisition and preprocessing procedures employed by the NEMO team to achieve these goals.

**Methods and analysis:**

To meet the study’s objectives, NEMO utilizes multiple imaging techniques, including T1-weighted structural MRI, resting-state fMRI, motor task fMRI, and 18F-FDG PET scans. We will outline our efforts over the past 4 years to enhance the quality of our collected data, and address challenges such as head movements during image acquisition, choosing acquisition parameters and constructing data preprocessing pipelines. This study is the first to employ these neuroimaging modalities in a standardized approach contributing to more uniformity in the analyses of future studies comparing these patient groups. The data collected will contribute to the development of a machine learning-based classification tool and improve our understanding of disorder-specific neurobiological factors.

**Ethics and dissemination:**

Ethical approval has been obtained from the relevant local ethics committee. The NEMO study is designed to pioneer the application of machine learning of movement disorders. We expect to publish articles in multiple related fields of research and patients will be informed of important results via patient associations and press releases.

## 1 Introduction

Hyperkinetic movement disorders are clinically characterized by excessive involuntary movements (Abdo et al., 2010). Three of the main phenotypes are essential tremor, dystonia and cortical myoclonus. Essential tremor has a prevalence of 0.9% in the general population and incidence increases with age to 4.6% in the population older than 65 years (Louis and Ferreira, 2010). This phenotype is characterized by rhythmic and sinusoidal alternating movements (Bhatia et al., 2018). Dystonia is defined as sustained or intermittent muscle contraction causing abnormal, often repetitive movements, postures, or both, and has a prevalence of 16 per 100.000 (Steeves et al., 2012). Myoclonus, defined as sudden, brief shock-like movements, has a prevalence of 9 per 100.000 (Caviness et al., 1999). These disorders often severely limit patients in their daily lives (Chandran and Pal, 2013; van der Stouwe et al., 2015), and, because of the visibility of the disorder, patients are prone to embarrassment and social isolation (Chandran and Pal, 2013; Cullinane et al., 2014; Van der Stouwe et al., 2015).

It is important to classify the movement disorder phenotype correctly, as this determines the subsequent diagnostic and treatment process that will be initiated for a patient (Brandsma et al., 2021; van der Veen et al., 2021; Sadnicka and Edwards, 2023). For example, in dystonia it is important for the diagnosis if a patient has isolated dystonia or dystonia with tremor and/or myoclonus, as this can provide clues about the underlying etiology, i.e., by which disease the dystonic phenotype is caused. In addition, treatment in hyperkinetic movement disorder patients is mainly symptomatic and differs per phenotype. For example, botulinum toxin injections are effective in dystonia, sometimes in dystonic tremor, but almost never in isolated tremor. In patients with medically refractory movement disorders, deep brain stimulation (DBS) has become a preferred treatment (Krack et al., 2019). Importantly, the optimal brain target for DBS primarily depends on phenotype, underscoring the need for objective phenotyping.

Currently, clinical classification of involuntary movements (i.e., phenotyping) is purely based on clinical definitions and thus on expert opinion (Abdo et al., 2010). However, there is large inter- and intra-observer variability during phenotyping (Van der Salm et al., 2013; Beghi et al., 2014; Eggink et al., 2017; Van der Salm et al., 2017). This is a major problem, which impairs patient diagnostics, evaluation of disease progression, personalized treatment, and treatment monitoring. To solve this problem, the Next Move in Movement Disorders (NEMO) project was set up by the Department of Neurology, University Medical Center Groningen (UMCG) in 2018. The aims of this project are twofold: firstly, to develop a computer-aided classification tool that can support neurologists to quickly and confidently arrive at phenotype classification of hyperkinetic movement disorders, and secondly, to better understand the pathophysiology of these hyperkinetic movement disorders.

**Figure 1:**
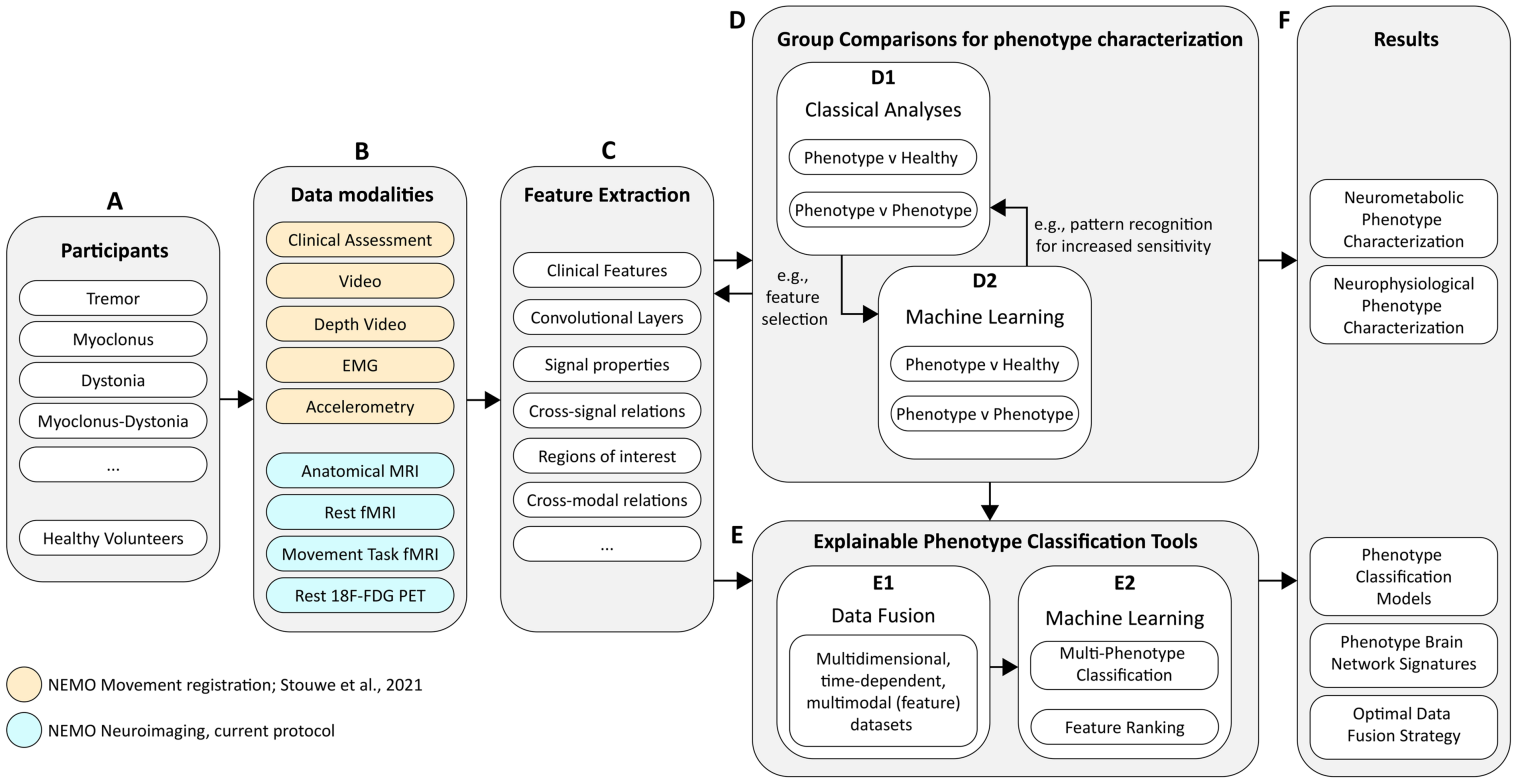
Schematic diagram of the technical approach for project NEMO. The diagram covers **(A)** the study participant groups, **(B)** the data modalities of all measurements, **(C)** the planned approaches for feature engineering, **(D)** phenotype characterization and **(E)** phenotype classification, and **(F)** the desired project results.

The technical approach of NEMO is shown in the diagram illustrated in Figure 1. Currently, we are recruiting adult tremor, myoclonus, dystonia, and myoclonus-dystonia patients (Figure 1A, see section 2) but also plan to extend this work to, e.g., more complex and mixed movement disorders, such as functional movement disorders and movement disorders caused by cerebral palsy. To achieve our aims, we acquire data using movement registration and neuroimaging measurements (Figure 1B). The methodology regarding the study setup and movement registration is further explained in detail by Van der Stouwe et al. (2021). The current protocol review is focused on the neuroimaging modalities of NEMO and describes the methods used for the functional magnetic resonance imaging (fMRI) and ^18^F-fluorodeoxyglucose positron emission tomography (^18^F-FDG PET) measurements of the brain. From each data modality, we estimate and *extract features* (Figure 1C) that we use for analyses to gain insights into how hyperkinetic movement disorders affect brain metabolism and to perform comparative analyses among disorders to improve our understanding of their distinctive attributes. These comparative analyses will involve (a) *group comparisons for phenotype characterization* (Figure 1D1), in which we try to find and understand group differences in brain metabolism during rest and during motor action, and (b) machine learning approaches to assist neurologists with *explainable phenotype classification* (Figure 1D2). For phenotype characterization, we will consider analyses that are classically used in the respective research domains, which we will supplement with machine learning approaches such as multivoxel pattern analyses to increase sensitivity for finding group differences (Haxby, 2012). Classical analyses may also provide multimodal feature inputs for these machine learning approaches. For example, ^18^F-FDG PET group comparison results may be used to define regions of interest for analyses in the fMRI data or vice versa.

In addition to phenotype characterization, we plan to *fuse data* (Figure 1E1) from different modalities to identify which complementary data modalities result in the best prediction performance and to determine which data modalities are most efficient in terms of costs and benefits. Our ultimate goal is to build *explainable phenotype classification tools* (Figure 1E2). For the adoption of such technology in clinical practice, it is imperative that machine learning “decision-making” is insightful and a transparent guiding tool for clinical experts. We plan to employ machine learning systems that are either genuinely interpretable, such as prototype-based techniques, or combine machine learning with tools that provide interpretable explanations for complex models (Biehl et al., 2016; Barredo Arrieta et al., 2019; Van et al., 2022). The models need to be supplemented with *feature ranking* (Figure 1E2) techniques that show where—in time and space—important phenotyping information is present and why it is important. Initially, these models will be developed for classifying clear and isolated hyperkinetic movement disorders, as a first step toward classifying more complex and mixed movement disorder phenotypes.

## 2 Study population

### 2.1 Recruitment

Neuroimaging data is currently being acquired from 20 dystonia, 20 essential tremor, 20 cortical myoclonus, and 40 mixed movement disorders patients (i.e., myoclonus-dystonia) in whom the disorder affects hand or arm function. Furthermore, 40 age- and sex-matched healthy participants are being recruited for comparison. Patients are selected from the UMCG hyperkinetic movement disorders database, and are recruited mainly at the UMCG outpatient clinics, with 10 patients via other hospitals in the Netherlands, patient associations, and patient research platforms. Healthy participants are recruited via the UMCG. Participants receive written information about the study and have the opportunity to ask the investigators questions beforehand. Participants are included if they are 16 years of age or older. Exclusion criteria are: (Abdo et al., 2010) other neurological conditions that lead to movement problems other than the hyperkinetic movement disorder, (Louis and Ferreira, 2010) other conditions that lead to impaired hand or arm function, and (Bhatia et al., 2018) any contraindications for MRI. In addition, healthy volunteers who are first-degree relatives of patients with movement disorders are excluded as well. For the current protocol review, patients or the public were not involved in the design, or conduct, or reporting, or dissemination plans of our research. They will be involved in follow-up studies that will report on the NEMO study results.

### 2.2 Sample size

Estimating the necessary sample size for machine learning studies is challenging. Unlike hypothesis testing, where power analysis is common, machine learning focuses on assessing the model’s generalizability through validation on independent data sets. For our study, we selected 20 subjects per group based on relevant literature and our experience, believing this number would be sufficient to develop initial machine learning classifiers for clear and isolated hyperkinetic movement disorders. Phenotypes. The group sizes were realistic given the rarity of these disorders and the fact that a number of patients had to be excluded since they had already undergone deep brain stimulation or had contraindications for MR imaging. Despite our clinic being one of the few rare movement disorder expertise centers in the in the Netherlands reaching patients across the country, we anticipated that larger group sizes were not feasible.

To determine if our sample sizes were sufficient to detect differences between groups, we conducted a power calculation using NeuroPowerTools (Durnez et al., 2016).^1^ This calculation was based on brain maps from two groups: 21 essential tremor patients performing a postural task and 21 healthy participants mimicking tremor during the same task (Van Der Stouwe et al., 2016). With a significance level (alpha) of 0.05 (corrected) and a desired power of 0.80, the Bonferroni correction indicated that the between-group comparison achieved a power of 0.87. Based on these results, we concluded that having 20 participants per group would be sufficient for our planned neuroimaging group comparisons.

### 2.3 Ethics and dissemination

The study was approved by the medical ethical committee of the UMCG (METc 2018/444) and written informed consent is obtained from all subjects according to the Declaration of Helsinki. Given the scope of this study, we expect to publish multiple articles in the fields of neuroimaging, clinical neurology, particularly movement disorders, clinical neurophysiology, artificial intelligence and visual analytics. Moreover, patients will be informed of important study results via the different patient associations, press releases, the website www.movementdisordersgroningen.com and at biannual Movement Disorders Groningen Patient Days.

## 3 Methods and analysis

### 3.1 fMRI acquisition strategies

Hyperkinetic movement disorders are often mild or non-existent during rest but become more pronounced during postural or kinetic tasks (Abdo et al., 2010; Jinnah and Factor, 2015; Bhatia et al., 2018; Roze et al., 2018). Therefore, we aimed to measure brain function both in rest and during a motor task to evoke the disorders. As a result, one of the main challenges for the neuroimaging part of the NEMO project was accounting for the increased risk of involuntary movement-induced artifacts during the measurements, as removing the artifacts is crucial to properly studying the true neurobiological signals (Power et al., 2018). To mitigate this risk, we took extra care in designing the neuroimaging protocols.

FMRI measures blood oxygen level dependent (BOLD) signals. However an (f) MRI signal is sensitive to body movements resulting in motion artifacts and thereby the reduction of signal to noise ratios (SNR) in the data. For fMRI studies in patients with movement disorders, this means that a greater number of trials is needed to produce data of sufficient quality thus limiting the number of experimental variables one can introduce. This constrains the hypotheses that can be tested. Fortunately, advances in MRI hardware and analysis tools allow for creating fMRI protocols that are more robust against such movement-induced signal artifacts.

**Figure 2:**
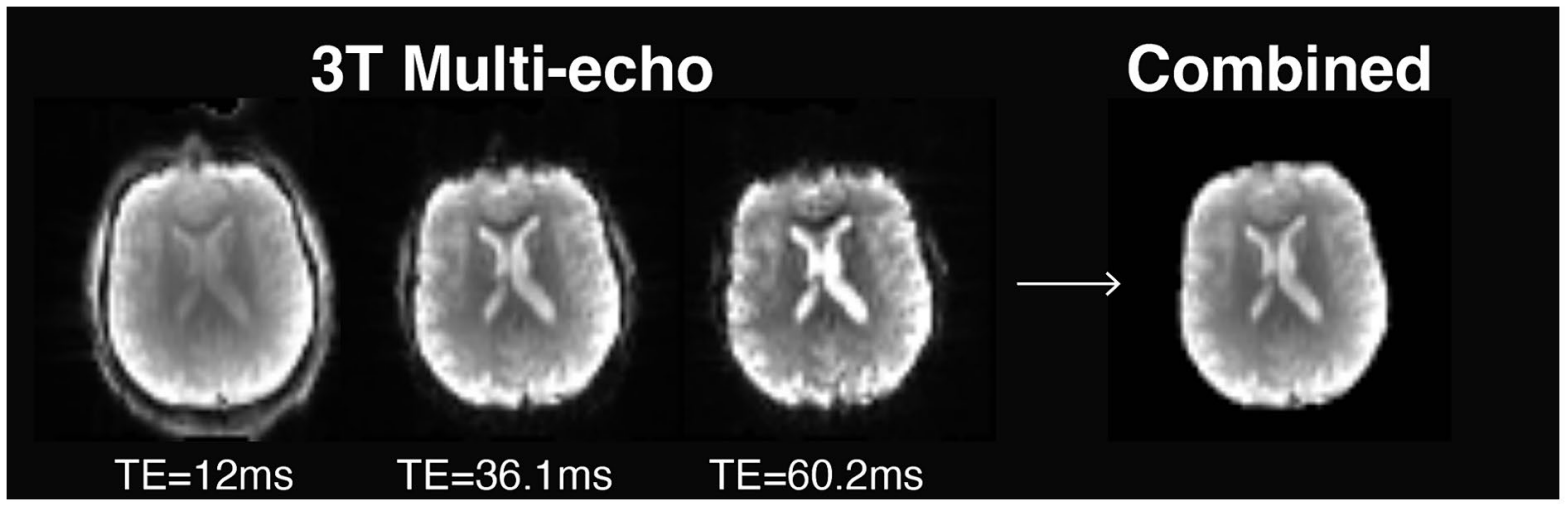
Multi Echo data acquisition at different echo times (TE).

While setting up (f) MRI protocols for NEMO, we evaluated the use of multi-echo (ME) fMRI during hand movement tasks and resting state (i.e., absence of a stimulus or a task). In conventional fMRI sequences, data of an image is collected following a radio frequency pulse within a time period denoted as echo time. This type of imaging is known as single-echo fMRI. For ME fMRI, multiple brain images are collected following each radio frequency pulse at different echo times. Figure 2 shows how signal intensity decays over time as a function of echo time on a 3 T MRI scanner. In some areas, signal intensity fully decays, a phenomenon called ‘signal dropout’, which is caused by inhomogeneities in the main magnetic field (Glover, 2011). By using data across multiple echo times, the images can be combined into an optimal brain image. This strategy not only improves spatial image quality by recovering signal loss in dropout areas, but also offers a twofold increase in temporal SNR for BOLD fMRI (Kundu et al., 2013).

The relationship between signal strength and echo time may also be exploited to assess underlying signal sources. By analyzing signal change across time points and echo times, BOLD (neuronal) and non-BOLD (artifactual) signal components can be distinguished using an approach that combines ME fMRI imaging with independent component analysis (ME-ICA). In this context, non-BOLD components, such as movement-induced artifacts, are removed from the data, resulting in a fourfold improvement in BOLD SNR (Kundu et al., 2013; Olafsson et al., 2015; Kundu et al., 2017; DuPre et al., 2021).

A common challenge for constructing (ME) fMRI protocols is choosing a set of parameters affecting the spatial and/or temporal resolution of the measurements. To improve the resolution, (ME) fMRI protocols generally make use of acceleration techniques for data acquisition. Two common fMRI acceleration techniques are GeneRalized Autocalibrating Partially Parallel Acquisition [GRAPPA, also termed iPAT, (Griswold et al., 2002)], and partial Fourier (Feinberg et al., 1986). GRAPPA allows acquiring less data to construct brain volumes by integrating spatial information from reference volumes that are obtained prior to the scan. The advantage of this acceleration technique is that it reduces both image distortion and drop out effects (Schmiedeskamp et al., 2010). However, the use of reference scans makes this method more susceptible to head motion; when subjects move during reference scans, the entire time-series will be affected.

A different approach to increase the spatio-temporal resolution is partial Fourier. Like GRAPPA, partial Fourier acquires less data per volume but does not require reference volumes. Instead, partial Fourier mathematically synthesizes missing data from the volumes themselves. The strength of partial Fourier compared to GRAPPA, is that it is less sensitive to motion. However, partial Fourier has slightly lower spatial SNR (resulting in less sharp images) and is more prone to signal drop out, especially in the frontal cortex and lateral temporal lobes (Smith et al., 2013).

In addition to GRAPPA and partial Fourier, data acquisition may be accelerated using multiband (MB, also termed simultaneous multi slice, SMS) acceleration. Conventional fMRI sequences acquire data slice-by-slice to create 3D volumes. Multiband fMRI makes use of a multiband radiofrequency pulse that simultaneously excites and receives signals from multiple slices reducing the time needed to acquire brain volumes [i.e., time repetition (TR)]. The benefit of shortening the TR in the context of hyperkinetic movement disorders is that it allows for better sampling of (movement induced) artifacts. However, multiband acceleration also leads to spatially heterogeneous noise amplification reducing SNR. The recommended balance between costs and benefits is an acceleration factor of MB = 4 on a 3 T MR scanner, reflecting a four-fold increase in the rate that brain volumes are acquired (Risk et al., 2021).

To measure BOLD fMRI in patients with hyperkinetic movement disorders, and acknowledging the increased risk of motion-induced artifacts while scanning, we aimed to build two protocols with different trade-offs affecting spatial and temporal signal quality. For the first acquisition protocol, we aimed to make relatively small sacrifices in spatial and temporal signal quality and created a full brain T2*-weighted echo-planar imaging (EPI) protocol that combined Partial Fourier acceleration and multiband imaging with an isotropic voxel resolution of 2 mm and a temporal resolution of 1,600 ms.

The second protocol involved hand movement, and thus we expected more movement related artifacts. Therefore, we aimed to make the second acquisition protocol more robust against head movement induced artifacts and chose for a shorter TR to enhance artifact sampling, using multi-echo imaging to separate BOLD from artifact signal sources using ME-ICA, and relatively large voxels as larger voxels are less sensitive to motion artifacts (Risk et al., 2021). To this end, we created a full brain T2*-weighted EPI protocol that combined Partial Fourier acceleration, multi-echo, and multiband imaging with an isotropic voxel resolution of 3.5 mm and temporal resolution of 1,101 ms.

### 3.2 fMRI protocols

Magnetic resonance imaging (MRI) data are collected on a 3 T Siemens Prisma scanner at the UMCG using a Siemens 64-channel head coil. T1-weighted sagittal images (MPRAGE) are acquired at 1 mm isotropic resolution with the following parameters: TR = 2,300 ms; TE: 2.98 ms; FA = 9°; 256 slices; Bandwidth = 240 Hz/Px. Functional MRI data is being acquired using two protocols: a multiband as well as a multiband multi-echo protocol. The multiband protocol consists of a full brain T2*-weighted echo-planar sequence with scanning parameters: TR = 1,600 ms; TE = 34 ms; FOV = 224 mm; FA = 70°; voxel size = 2 mm isotropic; 72 slices; Partial Fourier = 6/8; MB = 4; Bandwidth = 1828 Hz/px; Pulse duration = 5,120 μs; AP phase encoding direction; MB LeakBlock kernel enabled; EPI factor = 114. The multiband multi-echo protocol consists of a full brain T2*-weighted echo-planar sequence with scanning parameters: TR = 1.101 ms; TE = 12, 36.1, 60.2 ms; FA = 45°; voxel size = 3.5 mm isotropic, 48 slices, Partial Fourier = 6/8 (no IPAT), MB = 4; bandwidth = 2,604 Hz/px; pulse duration = 2,560 μs; AP phase encoding direction, MB LeakBlock kernel enabled; EPI factor = 64. In addition, 10 additional volumes with inverted RO/PE polarity are acquired for each protocol for distortion correction purposes. The EPI pulse sequence used for both protocols was generously provided by the Center for Magnetic Resonance Research (CMRR) at the University of Minnesota (Xu et al., 2013).

### 3.3 fMRI tasks and procedures

To assess spatial and temporal signal quality of the fMRI protocols during rest and hand movements, the first 23 participants underwent an extended fMRI session comprised of two resting-state and three task T2*-weighted fMRI scans, and one T1-weighted anatomy scan. Resting-state was measured once using the multi-echo fMRI protocol (9 min 54 s) and once using the single-echo (9 min 52 s) (“rest1” & “rest2,” respectively). For the resting-state scans, participants were instructed to lie motionless in the scanner, fixate their eyes on a white fixation cross on a black screen, and let their mind wander freely.

Since hyperkinetic movement disorder phenotypes become more pronounced during postural or kinetic tasks, we measured brain responses during two different hand movement tasks. The first hand movement task was a postural task in which participants were instructed to keep their arms pronated and outstretched with extended wrists (“hands1”). The second hand movement task was a kinetic self-paced four-finger tapping task in which participants had to tap their fingertips simultaneously on the thumb repeatedly, which was performed once during single-echo fMRI and once during multi-echo fMRI measurement (“hands2” & “hands3”). Brain responses during this task were only measured using the single-echo fMRI protocol. We chose a postural and a kinetic task as we were not sure if a kinetic hand movement task would cause too much head movements.

**Figure 3:**
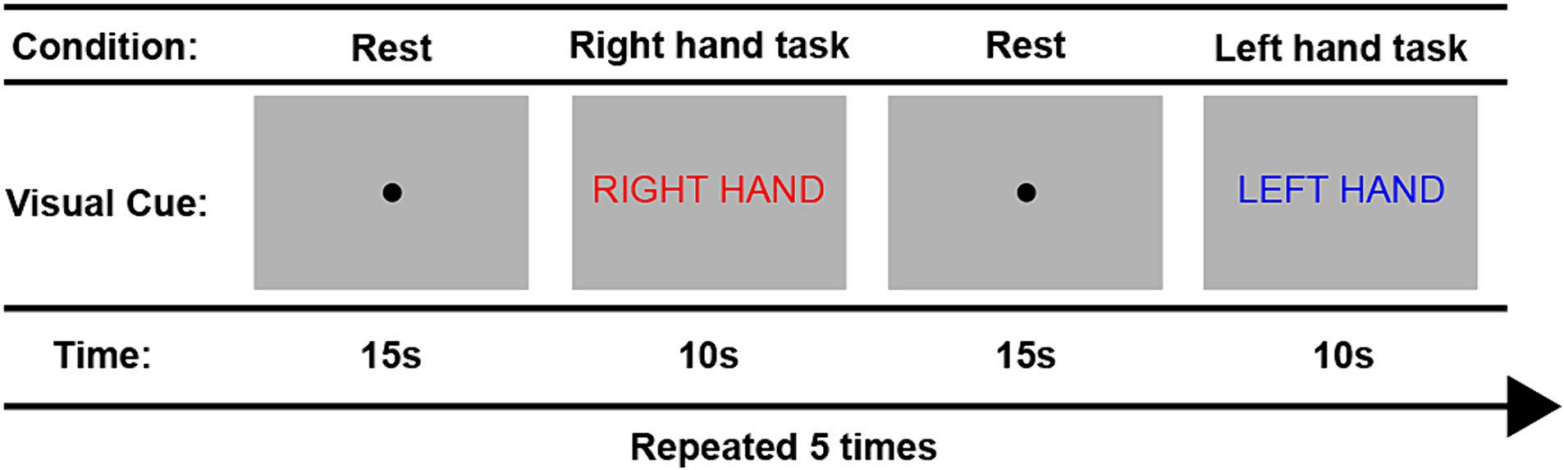
Overview of the fMRI finger tapping task paradigm.

Figure 3 provides a schematic overview of the fMRI task paradigms. Tasks were alternated between the right and left hand during 10 trials. Throughout the tasks, participants received visual cues and instructions in Dutch provided on a computer screen. Each trial began with the message “Right hand” (in Dutch: “Rechter hand” displayed in red font) or “Left hand” (in Dutch: “Linker hand” displayed in blue font) to indicate the corresponding hand movement block for the right and left hand, respectively. Within each block, participants performed the assigned hand movement task for a duration of 10 s. The hand used for tapping alternated between left and right, repeating a total of five times. A 15-s rest condition, indicated by a black dot, separated each hand tapping condition. The entire task lasted for 4 min and 43 s. To ensure accurate task performance, participants watched an instruction video prior to the scan and practiced the protocol inside the scanner before measurements began. Furthermore, we recorded task performance on video during the scan to later inspect data quality and ensure task compliance.

After assessing the protocols in 23 participants (see section 6), we condensed the fMRI session to one resting-state scan using the single-echo fMRI protocol, one kinetic hand movement task fMRI scan using the multi-echo fMRI protocol, and one anatomy scan.

### 3.4 fMRI preprocessing

#### 3.4.1 Anatomical data preprocessing

T1-weighted (T1w) images are corrected for intensity non-uniformity (INU) with N4BiasFieldCorrection (Tustison et al., 2010), distributed with ANTs 2.3.3 (Avants et al., 2008), and used as T1w-reference throughout the workflows. The T1w-reference was then skull-stripped with a *Nipype* implementation of the antsBrainExtraction workflow (ANTs 2.3.3), using OASIS30ANTs as target template. Brain tissue segmentation of cerebrospinal fluid (CSF), white-matter (WM) and gray-matter (GM) was performed on the brain-extracted T1w using fast [FSL 6.0.5, (Zhang et al., 2001)]. Volume-based spatial normalization to two standard spaces (MNI152nLin2009cAsym, MNI152nLin6Asym) was performed through nonlinear registration with antsRegistration (ANTs 2.3.3), using brain-extracted versions of both T1w reference and the T1w template.

#### 3.4.2 Multiband fMRI

The multiband data was preprocessed using fMRIPrep v20 (Esteban et al., 2019). The following methodology was used; first, a reference volume and its skull-stripped version were generated using custom methodology of *fMRIPrep*. A B0-nonuniformity map (or *fieldmap*) was estimated based on two EPI references with opposing phase-encoding directions, with 3dQwarp (Cox and Hyde, 1997; AFNI 20160207). Based on the estimated susceptibility distortion, a corrected EPI reference was calculated for a more accurate co-registration with the anatomical reference. The BOLD reference was then co-registered to the T1w reference using flirt (FSL 5.0.9; Jenkinson and Smith, 2001) with the boundary-based registration cost-function (Greve and Fischl, 2009). Co-registration was configured with nine degrees of freedom to account for distortions remaining in the BOLD reference. Head-motion parameters with respect to the BOLD reference (transformation matrices, and six corresponding rotation and translation parameters) are estimated using mcflirt (FSL 5.0.9; Jenkinson et al., 2002) before any spatiotemporal filtering. BOLD fMRI timeserieswere slice-time corrected to 0.745 s (half of slice acquisition range) using 3dTshift from AFNI 20160207 (Cox and Hyde, 1997). The BOLD time-series (including slice-timing correction when applied) were resampled onto their original, native space by applying a single, composite transform to correct for head-motion and susceptibility distortions. These resampled BOLD time-series will be referred to as *preprocessed BOLD*. The BOLD time-series were resampled into MNI space, generating a preprocessed BOLD time series in MNI space. Automatic removal of motion artifacts using independent component analysis [ICA-AROMA; Pruim et al. (2015)] was performed on the preprocessed BOLD on MNI space time-series after removal of non-steady state volumes and spatial smoothing with an isotropic, Gaussian kernel of 6 mm FWHM (full-width half-maximum). Thus, “non-aggresively” denoised BOLD timeseries were produced. Gridded (volumetric) resamplings were performed using antsApplyTransforms (ANTs), configured with Lanczos interpolation to minimize the smoothing effects of other kernels (Lanczos, 1964). Non-gridded (surface) resamplings were performed using mri_vol2surf (FreeSurfer).

#### 3.4.3 Multiband multi-Echo fMRI

Multiband multi-echo fMRI data was preprocessed using an in-house constructed nipype pipeline, which consisted of fMRIprep v22, TE-dependence analysis (tedana) v0.0.12, and Advanced Normalization Tools (ANTs) v2.3.5 (Avants et al., 2008; Gorgolewski et al., 2011; Esteban et al., 2019; DuPre et al., 2021). Here, fMRIPrep v22 was used instead of the long-term support version v20 since v22 provides enhanced functionalities for multi-echo fMRI that better support post-processing with tedana. First, fMRIprep preprocessing was applied to the data using the “single-echo” output parameter. The following methodology was used; first, a reference volume and its skull-stripped version were generated from the shortest echo of the BOLD timeseries using custom methodology of *fMRIPrep*. A *B_0_*-nonuniformity map (or *fieldmap*) was estimated based on two EPI references with topup (FSL 6.0.5.1; Andersson et al., 2003; Graham et al., 2017). Head-motion parameters with respect to the BOLD reference (transformation matrices, and six corresponding rotation and translation parameters) are estimated before any spatiotemporal filtering using mcflirt (FSL 6.0.5.1; Jenkinson et al., 2002). The estimated *fieldmap* was then aligned with rigid-registration to the target EPI reference volumes. The field coefficients were mapped on to the reference EPI using the transform. BOLD timeseries were slice-time corrected to 0.495 s (half of slice acquisition range) using 3dTshift from AFNI (Cox and Hyde, 1997). A T2* map was estimated from the preprocessed EPI echos, by voxel-wise fitting the maximal number of echoes with reliable signal in that voxel to a monoexponential signal decay model with nonlinear regression. The T2*/S0 estimates from a log-linear regression fit were used for initial values. The calculated T2* map was then used to optimally combine preprocessed BOLD across echoes following the method described in (Posse et al., 1999). The optimally combined time series was carried forward as the *preprocessed BOLD*. The BOLD reference was then co-registered to the T1w reference using mri_coreg (FreeSurfer) followed by flirt (FSL 6.0.5.1; Jenkinson and Smith, 2001) with the boundary-based registration cost-function (Greve and Fischl, 2009). Co-registration was configured with six degrees of freedom. The same confounds and resampling were calculated as for the multiband fMRI protocol.

The fMRIPrep “single-echo” output parameter provided individual echo time series with slice, motion and susceptibility correction. These individual echo time series were used as input for multi-echo independent component analysis (ME-ICA) denoising, using Python library tedana (DuPre et al., 2021). A custom brain mask was used calculated from the BOLD-reference file generated by fMRIPrep. The resulting denoised BOLD time series was normalized to MNI space with ANTs using the transformation file generated by fMRIPrep, and finally resliced to 2 mm isotropic voxels.

### 3.5 fMRI task and protocol evaluation

**Figure 4:**
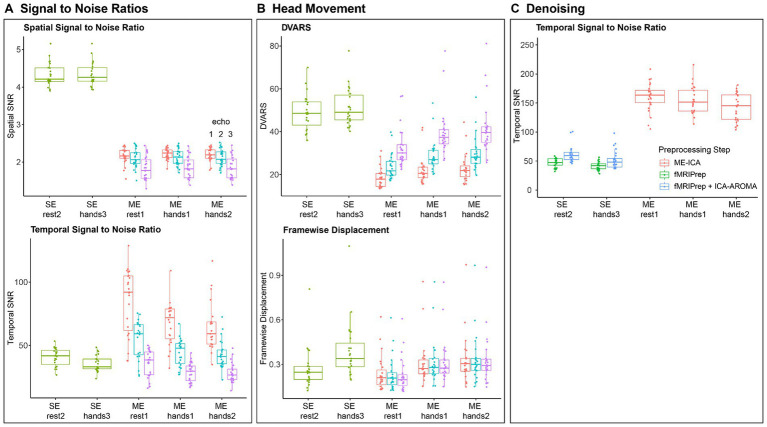
Signal Quality Metrics across fMRI Protocols and Tasks. **(A)** Spatial and Temporal signal to noise (SNR) metrics for both fMRI protocols across all tasks before preprocessing. **(B)** Head movement metrics for both fMRI protocols across all tasks. We show DVARS (D referring to temporal derivative of time courses, VARS referring to RMS variance over voxels) and Framewise Displacement (Power et al., 2012). Metrics were calculated for gray matter voxels using MRIQC (Esteban et al., 2017). **(C)** Temporal SNR for the single-echo and multi-echo protocol after fMRIPrep preprocessing, ICA-AROMA denoising (Pruim et al., 2015) and ME-ICA denoising (Kundu et al., 2013; DuPre et al., 2021).

To assess spatial and temporal signal quality for the two fMRI protocols across rest and hand movement tasks, we compared data quality metrics in a small cohort of hyperkinetic movement disorder patients (1 dystonia, 7 myoclonus, 2 tremor) and 13 healthy controls using framewise displacement (FD), derivative of the root mean square variance over voxels (DVARS), spatial SNR, and temporal (t) SNR estimated by MRIQC [48, 49]. The results are shown in Figure 4 and Table 1.

**Table 1:** Average signal quality metrics across fMRI protocols and tasks.

Sequence	Single-echo		Multi-echo								
Task	rest2	hands3	rest1			hands1			hands2		
Echo time (ms)	34	34	12	36.1	60.2	12	36.1	60.2	12	36.1	60.2
Spatial SNR	4.33 (0.32)	4.34 (0.31)	2.17 (0.22)	2.10 (0.25)	1.83 (0.31)	2.22 (0.15)	2.13 (0.22)	1.85 (0.30)	2.20 (0.17)	2.12 (0.23)	1.85 (0.30)
Temporal SNR	40.81 (6.96)	35.64 (6.34)	84.94 (26.17)	54.76 (15.19)	35.79 (10.40)	67.48 (17.25)	44.25 (10.74)	28.58 (7.63)	64.35 (19.04)	42.97 (11.14)	28.13 (7.83)
FD	0.27 (0.14)	0.39 (0.19)	0.24 (0.12)	0.23 (0.12)	0.23 (0.12)	0.31 (0.16)	0.32 (0.16)	0.32 (0.16)	0.33 (0.17)	0.34 (0.17)	0.33 (0.17)
DVARS	48.90 (8.52)	51.33 (8.85)	18.38 (4.75)	23.85 (6.07)	32.66 (9.32)	22.21 (6.85)	28.82 (7.65)	39.80 (11.39)	23.18 (6.88)	29.95 (8.24)	41.80 (12.59)

All values are expressed in mean and (sd). Ms, milliseconds; SNR, signal to noise ratio; FD, framewise displacement; DVARS, derivative of the root mean square variance over voxels.

Our aims were to compare (Abdo et al., 2010) spatial SNR across protocols, (Louis and Ferreira, 2010) head movements and head movement related artifacts across movement and rest tasks, and whether these differed between patients and controls, and (Bhatia et al., 2018) temporal SNR across protocols and tasks after preprocessing and denoising. Statistical comparisons were performed in R 4.3.1^2^ using mixed effect models (LME4 1.1–35.1 and LmerTest 3.1–3).

To investigate differences in spatial SNR across protocols (Figure 4A), we constructed a mixed effect model with spatial SNR as dependent variable, protocol (single-echo vs. multi-echo) as independent variable and participant ID as random variable. We found that the single-echo protocol had 2.12x higher spatial SNR than the multi-echo protocol [*t* (229) = 73.85, *β* = 2.29, *p* < 0.001].

To find differences in head movement related differences across tasks (Figure 4B), we constructed two models that tested for differences in head displacements and movement related noise variance (DVARS), respectively, as a function of the interaction between task type (movement vs. rest) and participant group (patient vs. control). Again, patient ID was used as random variable. We found that head displacements were 1.34x larger [*t* (228.02) = 11.98, *β* = 0.097, *p* < 0.001] and that DVARS was 1.11x larger during hand movement tasks [*t* (228.12) = 4.92, *β* = 5.37, *p* < 0.001]. We found no differences between patients and controls (all *p* > 0.5).

Finally, data quality after preprocessing and denoising (Figure 4C) was investigated using a mixed effect model with temporal SNR as dependent variable, the interaction between protocol type (single-echo vs. multi-echo) and task type (movement vs. rest) as independent variables, and patient ID as random variable. For single-echo data, preprocessing and denoising comprised of fMRIPrep combined with ICA-AROMA while fMRIprep combined with ME-ICA were used for the multi-echo data. The results showed that tSNR was 1.24x higher for the resting task compared to the movement task [*t* (24) = 2.92, *β* = 9.89, *p* < 0.01]. After ME-ICA denoising of the multi-echo data, we found that tSNR was 1.11x higher in the rest task compared to the hands2 task [*t* (47.22) = 3.47, *β* = 0.097, *p* < 0.01 corrected] but we found no difference between the rest1 and hands1 tasks. More importantly, tSNR was 2.69x higher for the denoised multi-echo data compared to the denoised single-echo data [*t* (98.34) = 26.33, *β* = 95.72, *p* < 0.001].

During the protocol assessment, we thus found that multi-echo fMRI provides an almost threefold increase in BOLD signal quality by removing (motion-induced) signal artifacts but that protocol adjustments come at a cost in spatial SNR; for our protocols, single echo fMRI provided an over twofold increase in spatial SNR. As we observed relatively little head movements during resting-state scans, we decided to continue measuring resting-state using the single-echo protocol for the remaining participants.

Since head movements were far greater during hand movement tasks compared to rest, we decided to continue measuring brain responses using the multi-echo protocol for the movement task to benefit from the almost threefold improvement in temporal SNR. For the hand movement task, we decided to continue measurements using the kinetic hand movement task (“Hands2”), since it contains both a postural and kinetic component to invoke a wider spectrum of movement disorders than a purely postural task while head displacement and noise variance were similar between both tasks.

### 3.6 [^18^F]FDG PET acquisition strategies

PET is an imaging technique that relies on the injection of a radioactive compound (i.e., radiotracer). Many different radiotracers have been developed to visualize different brain functions or pathologies *in vivo*. 18F-FDG is the most commonly used radiotracer in clinical practice. It is a radioactive analog of glucose and depicts glucose consumption in the brain and, therefore, measures brain metabolism. The main advantage of the use of PET is that it is a quantifiable imaging technique, allowing for a direct comparison of function (radiotracer uptake by certain brain regions) between patients and healthy volunteers. By having subjects undergo 18F-FDG PET scans at rest with their eyes closed, we acquire images that show default baseline brain function.

The main challenges to scan patients for NEMO using 18F-FDG PET are (Abdo et al., 2010) the fact that hyperkinetic movement disorders may be mild or non-existent at rest, (Louis and Ferreira, 2010) increased risk of movement induced measurement artifacts, and (Bhatia et al., 2018) partial volume effects.

Movement disorders have been hypothesized to be network disorders (Andersson et al., 2003; Gorgolewski et al., 2011; Graham et al., 2017). This means that instead of affecting specific aspects of the brain, they affect the connections between brain regions. At rest, the combination of active regions that keep basic functions of the body at work is called the default mode network (Posse et al., 1999). While the histopathological hallmark of movement disorders is located in the basal ganglia, the effects of these diseases can be observed within the cortex, in which the default mode network resides. We therefore expected specific patterns of abnormal brain function affecting multiple regions of the brain at rest.

Similarly to fMRI, PET is a technique that is sensitive to head movements during image acquisition. Head movements may reduce image resolution and distort uptake values (especially in smaller brain regions). For the NEMO PET protocol, we decided not to restrict head movement using a stereotactic device or a thermoplastic mask to avoid patient discomfort. Instead, to verify how much head movement was happening during the scan, we opted for a dynamic acquisition protocol, in which brain images are reconstructed every 2 min during a 10-min data acquisition, resulting in 5 images per subject. Head movements between these images are assessed per individual. To generate single static 18F-FDG PET images for further analysis, the 5 dynamic images were corrected for motion using a conservative frame-based image-registration (FIR) approach, and then averaged to form a single image (Power et al., 2012). This procedure was performed during reconstruction on the scanner.

In addition to head movement susceptibility, PET images have a limited spatial resolution compared to MRI. Resolution can be affected by the inherent physical effects such as detector size, positron range, and non-collinearity (Esteban et al., 2017). Partial volume effects are a consequence of low spatial resolution, which result in lower radioactive uptake measurement than expected. Advances in PET imaging technology are reducing these image resolution effects by reducing the size of the crystals in the detectors and by using corrections during reconstruction such as time-of-flight (Boellaard et al., 2010) and iterative imaging reconstruction approaches, e.g., ordered subsets expectation maximization (OSEM; Peretti et al., 2019). Post-reconstruction partial volume effects correction can also be performed using methods such as recovery coefficient (Peretti et al., 2021), geometric transfer matrix (Cox, 1996; Esteban et al., 2017), and the Muller-Gartner approach (Hoopes et al., 2022). However, there is no clear consensus on which method for correction should be used. Furthermore, partial volume correction has been shown to increase variance in the data (Isensee et al., 2019), leading to a larger biases when comparing patients and controls (Avants et al., 2011; Norgaard et al., 2022). As images were acquired on Siemens Biograph mCT PET/CT scanners, which are two of the most recent scanners from Siemens, providing high resolution images, combined with OSEM and time-of-flight correction reconstructions, we decided against using partial volume corrections during image processing in the NEMO project.

### 3.7 [^18^F]FDG PET protocol

Prior to the scan session, subjects were asked to fast for at least 6 h and not to perform any heavy physical activity. These requirements are made to guarantee a higher uptake of ^18^F-FDG to brain tissue and less in other tissues, such as muscles or organs in the digestive tract, ensuring high measurement signal to noise ratio in the brain. To confirm subject compliance to the fasting protocol, plasma glucose levels were measured before PET scans and only subjects with levels lower than 7 mmol/L were scanned (Boellaard et al., 2010).

After glucose measurement, a bolus injection of an average of 200 MBq ^18^F-FDG via intravenous catheter was performed. Next, a buffered saline injection (approximately 10 mL) was administered to flush out the cannula. Participants were left to rest awake in a silent room with dimmed lights for 30 min before image acquisition for the radiotracer to achieve a steady state throughout the body (i.e., the net flux of tracer through tissue is stable).

Images are acquired using a Siemens Biograph 40 or 64mCT PET/CT scanner at the Department of Nuclear Medicine and Molecular Imaging of the University Medical Centre Groningen. Both scanners are from the same manufacturer and generation, acquisition and reconstruction protocols are harmonized, and the calibration of the systems is done equally. Therefore, we expected no difference between data from both scanners, as shown in previous studies (Peretti et al., 2019, 2021). ^18^F-FDG was synthesized at the radiopharmacy facility at the Department of Nuclear Medicine and Molecular Imaging according to Good Manufacturing Practice.

Dynamic image acquisition lasts 10 min (5 × 2 min) and images are averaged into a single static scan. Data are acquired in list-mode format for all scans and reconstructed using a 3D OSEM (3 iterations 24 subsets, 2 mm FWHM Gaussian kernel) using point-spread function and time-of-flight corrections. Final images are 400 × 400 × 111 matrices, with isotropic 2 mm voxels.

### 3.8 [^18^F]FDG PET preprocessing

**Figure 5:**
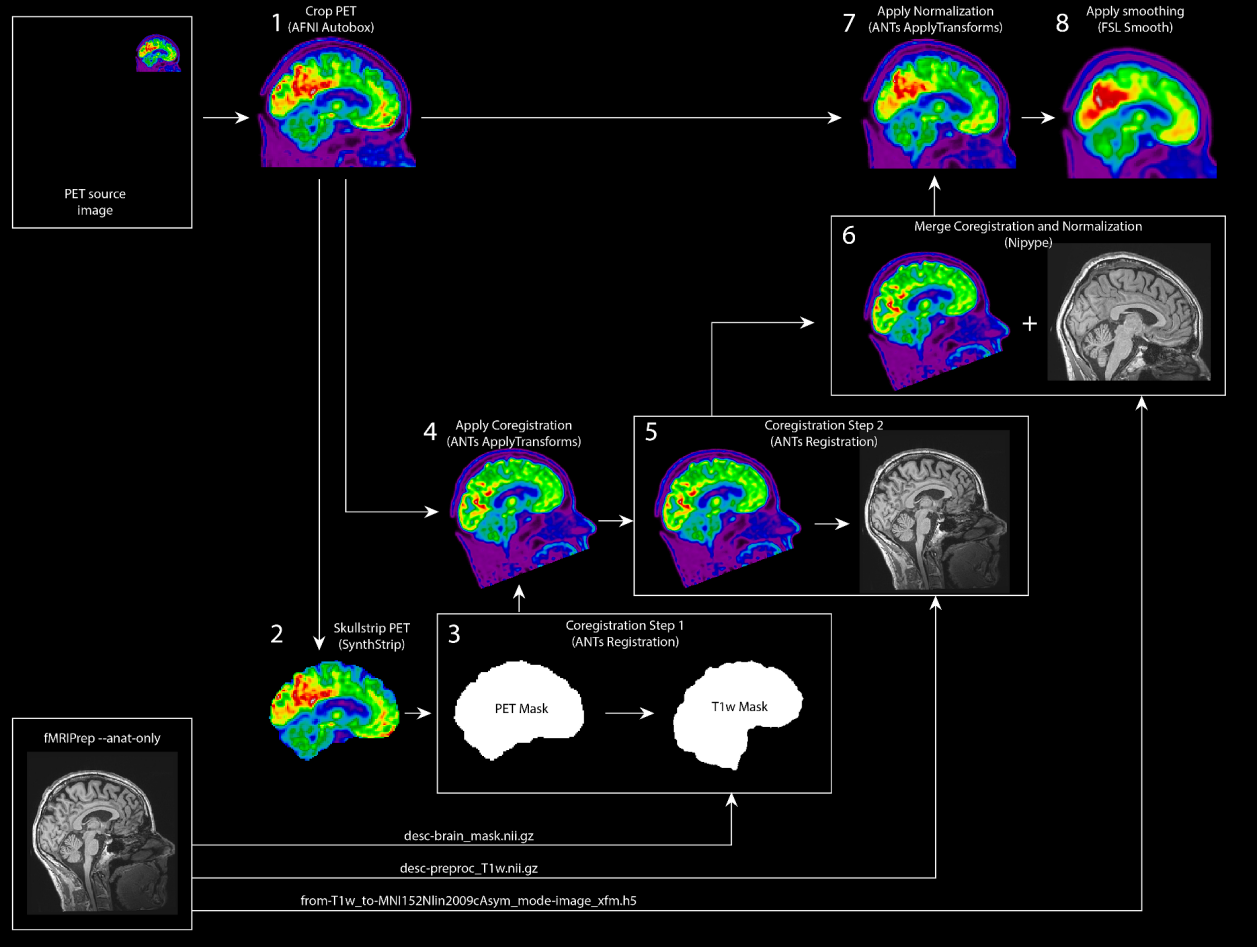
Robust FDG PET Preprocessing pipeline for project NEMO. Schematic overview of the preprocessing pipeline for the NEMO [18F] FDG PET images. As inputs, the pipeline uses a static PET input image and anatomical image preprocessing outputs from fMRIPrep. The PET image is skullstripped and subsequently coregistered to the anatomical image using two intermediate registration steps. The image registration transformations are merged with the normalization warping parameters from fMRIprep and then applied to the original PET image for a final PET image in MNI space.

For preprocessing the [^18^F]FDG PET images, we built a robust in-house preprocessing pipeline. First, the required anatomical images acquired during the MRI session, were preprocessed in fMRIPrep version 20.2.0 (Esteban et al., 2019) to obtain bias-corrected anatomical images, a brain mask, and normalization warping parameters to transform images from subject space to MNI space. Subsequently, we set up a Nipype (v1.8.3; Gorgolewski et al., 2011) preprocessing pipeline for the PET images. First, we cropped PET images using the autobox function from AFNI (v21.3.04; Cox, 1996) with 10 voxels padding. Next, we used SynthStrip for brain extraction (Hoopes et al., 2022). After testing multiple brain extraction toolboxes (i.e., FSL bet, FSL bet2, AFNI 3dSkullStrip, HD-BET (Isensee et al., 2019), and SynthStrip), SynthStrip showed very fast and robust performance across all PET datasets during in-house testing. Since coregistering the PET images to the bias-corrected anatomical images required a large spatial translation, we coregistered the images in two steps using Advanced Normalization Tools (ANTs, v2.3.5; Avants et al., 2011); first, we coregistered the brain extraction mask from SynthStrip to the anatomical brain mask, followed by a more fine grained coregistration step where the PET image was aligned to the T1 image. This two-step procedure proved to be more robust than coregistering both images in a single step. For further analyses, the preprocessing pipeline provides preprocessed images in subject space and in MNI space (i.e., *MNI152NLin2009cAsym*). To generate PET images in subject space, linear transformations from both coregistration steps are merged and applied to the original PET image. To generate preprocessed PET images in MNI space, linear transformations are merged with the normalization warping parameters from fMRIprep and then applied to the original PET image. An overview of the preprocessing pipeline is shown in Figure 5. The NEMO FDG PET preprocessing pipeline is available as Python package PETBrainPreprocessing at https://github.com/jrdalenberg/PETBrainPreprocessing. This package offers an open source robust Nipype workflow for preprocessing PET BIDS brain data (Norgaard et al., 2022).

After the preprocessing, T1 and FDG PET images were loaded into PMOD (version 4.1; PMOD Technologies LCC). The Automated Anatomical Labeling (AAL) atlas (Tzourio-Mazoyer et al., 2002) was chosen to delineate volumes of interest (VOI) as it contains the parcellation of the cerebellar gray matter, a brain region that is of importance in movement disorders. Tissue probability maps resulting from the PETBrainPreprocessing pipeline were also used as input to align VOIs to each subjects’ anatomy and to restrict analyses to gray matter voxels only. To compare FDG PET uptake between participants, standardized uptake value ratios (SUVR) were calculated by dividing each image voxel by the average uptake in the cortical gray matter. This intensity normalization step is what allows PET images to be compared between individuals. VOIs were then overlaid on the SUVR FDG PET images and average uptake per volume was extracted for further data analyses. Alternatively, the normalized PET images (i.e., in MNI space) can be used to study differences across groups at a voxel level, provided that an appropriate intensity normalization step is included in the analysis.

### 3.9 Quality assurance and control for both fMRI and PET

We took several precautionary measures to assure high quality data during collection and preprocessing for subsequent analyses. First, we designed study-specific case report forms in which data quality was carefully assessed right after each scan acquisition. When scan quality was insufficient (e.g., movement artifacts in the T1-weighted scans or when a participant accidentally moved their head out of the field of view of the scanner), the anatomical scan was repeated.

Second, we took care in ensuring that the hand movement tasks were correctly performed during fMRI scans. We created an instruction video that is shown prior to the MRI scan session. Furthermore, the movement tasks are trained inside the scanner and fMRI measurement starts once the task is correctly performed. In addition, we built an MR-compatible night-vision video-camera that allows us to evaluate task performance in the dark MR room during the scans. Video recordings during task performance are also available as reference during data analysis.

Third, we built in-house routines in our preprocessing pipelines to evaluate data quality. For the fMRI acquisitions, we use fMRIPrep html-reports. In addition, for the task fMRI, we construct simple mass univariate analysis to model motor responses of the left and right hand movements, create glass brain fMRI BOLD overviews, and make sure we can observe brain responses in the ipsilateral cerebellum and contralateral motor cortex.

## 4 Significance

The NEMO project represents one of the most extensive investigations into rare movement disorders. Its distinctive contribution lies in the integration of PET and fMRI neuroimaging techniques alongside movement registration measurements. Notably, this study is the first to systematically apply these measurement modalities across multiple hyperkinetic movement disorders, contributing to a more standardized approach to compare these rare movement disorders in future studies.

After establishing the distinguishing features, data modalities, and models for these relatively pure movement disorder phenotypes, our next goals are (1) to evaluate in close collaboration with neurologists which modalities, features and models are most efficient to implement in clinical settings, (2) to build machine learning approaches to assist neurologists in phenotype classification, and (3) to move toward addressing more complex mixed movement disorder phenotypes.

## Data Availability

The raw data supporting the conclusions of this article will be made available by the authors, without undue reservation. fMRI data quality metrics are available at Mendeley Data. DOI: 10.17632/p8rg7mv4h9.1.

## Footnotes

^1^ https://github.com/neuropower/neuropower-core

^2^ https://www.R-project.org/
